# Association of metabolic and inflammation vulnerabilities with systemic lupus erythematosus: a prospective UK Biobank study

**DOI:** 10.3389/fimmu.2026.1819233

**Published:** 2026-05-08

**Authors:** Dongqi Zhou, Lisha Sun, Na Li, Yi Liang, Gaofeng Gan, Dazhou Liao, Qiu Chen

**Affiliations:** 1 Department of Endocrine, Hospital of Chengdu University of Traditional Chinese Medicine, Chengdu, Sichuan, China; 2 Department of Infectious Diseases, Hospital of Chengdu University of Traditional Chinese Medicine, Chengdu, Sichuan, China; 3 Department of Traditional Chinese Medicine, Taikang Hospital of Sichuan Province, Chengdu, Sichuan, China; 4 West China Hospital, Sichuan University, Chengdu, Sichuan, China; 5 Hospital of Chengdu University of Traditional Chinese Medicine, Chengdu, Sichuan, China

**Keywords:** inflammation vulnerability, metabolic malfunction, metabolic vulnerability, systemic lupus erythematosus, UK Biobank

## Abstract

**Background:**

Systemic lupus erythematosus (SLE) is a heterogeneous autoimmune disease influenced by complex metabolic and inflammatory pathways, but the impact of specific metabolic and inflammatory signatures, particularly metabolic vulnerability index (MVX), inflammation vulnerability index (IVX), and metabolic malnutrition index (MMX), on the incidence of SLE remains unclear.

**Methods:**

We evaluated the association of MVX, IVX, and MMX with the risk of incident SLE using data from 398,200 participants in the UK Biobank. MVX was constructed from IVX (calculated using glycA and small HDL particles) and MMX (calculated using citrate, isoleucine, leucine, and valine). The primary outcome was SLE based on the International Classification of Diseases, 10th Revision (ICD-10) codes. Risks were estimated using Cox proportional hazards models, while restricted cubic spline (RCS) analysis was applied to identify potential non-linear trends. Subgroup analyses were performed by demographics, comorbidities, and lifestyle factors. Sensitivity analyses included a 3-year lag exclusion, landmark analyses at 5 and 10 years, additional adjustment for C-reactive protein (CRP), the use of time-dependent covariates combined with stratification, and the use of data after multiple imputation. Additionally, the Fine-Gray competing risk model was employed treating all-cause mortality and CVD-specific mortality as competing events.

**Results:**

Over a median follow-up of 13.2 years, a per standard deviation (SD) increment in MVX was associated with a 44% higher risk of SLE (HR = 1.44, 95% CI: 1.28-1.62, P < 0.01). Participants in the highest tertile (T3) of MVX had an 89% increased risk compared to those in the lowest tertile (T1) (HR = 1.89, 95% CI: 1.35-2.63, P < 0.01). Similar but attenuated associations were observed for IVX and MMX; per SD increases in IVX and MMX were associated with HRs of 1.34 (95% CI: 1.17-1.54, P < 0.01) and 1.24 (95% CI: 1.14-1.36, P < 0.01), respectively, while T3 versus T1 HRs were 1.85 (95% CI: 1.25-2.74, P < 0.01) and 1.82 (95% CI: 1.42-2.33, P < 0.01). MVX consistently demonstrated a stronger association with SLE than the sub-indices IVX and MMX. Subgroup analyses indicated that the significant association persisted across most groups, with a notable effect observed in females (HR = 1.59, 95% CI: 1.39-1.81, P < 0.01). RCS analysis confirmed a linear dose-response relationship, with risk thresholds of 36 for MVX, 40 for IVX, and 45 for MMX; beyond these thresholds, the risk of SLE increases as the index rises. Sensitivity analyses and the Fine-Gray competing risk model both confirmed the robustness of the study results, demonstrating that MVX is significantly associated with the risk of incident SLE.

**Conclusions:**

MVX serves as an independent risk factor for the development of SLE. Notably, MVX demonstrates a stronger association with SLE risk compared to its individual components, IVX and MMX. Our results suggest that MVX is valuable for risk assessment for SLE, particularly within the female population.

## 1 Introduction

Systemic Lupus Erythematosus (SLE) represents a chronic, refractory autoimmune entity distinguished by its predilection for the female population and its propensity to inflict multi-system damage (1). Influenced by geographic and demographic factors, the global incidence of SLE ranges from 0.3 to 23.2 cases per 100,000 person-years (2). Clinical hallmarks typically include musculoskeletal pain, fatigue, and mucocutaneous lesions (3, 4). Although the precise pathogenesis of SLE remains to be fully elucidated, the current consensus attributes the core pathological mechanisms to aberrant complement activation, immune complex deposition, and antibody dysregulation (5). Genetic polymorphisms, exogenous triggers, endocrine dysregulation, and aberrant immune responses play pivotal roles in the onset and progression of the disease (6). The prevailing diagnostic framework employs a positive ANA status as the entry criterion, supplementing it with a comprehensive weighted assessment that encompasses multi-organ clinical features and specific immunological indices (7, 8). Notwithstanding these advances, the precise quantification of disease activity remains a formidable challenge attributable to the intricate multi-system nature of SLE (9). Consequently, the development of novel assessment tools based on precision medicine is of paramount importance for optimizing early identification, prognostic evaluation, and the formulation of individualized management strategies for SLE.

Circulating metabolites are formed through the complex interaction between endogenous physiological processes and exogenous lifestyle factors and possess the potential to serve as predictive biomarkers for various health conditions (10, 11). With the continuous innovation and maturation of metabolomics technology, its potential in the discovery of new biomarkers for SLE has become increasingly prominent. Currently, there have been related studies reporting the use of metabolomics to assist in the development of new tools for the diagnosis and assessment of SLE (12–14). Despite these methods, investigating the potential association between practically accessible and simple metabolic indices with SLE still holds clinical significance. Recently developed, the metabolic vulnerability index (MVX) integrates plasma biomarkers quantified by Nuclear Magnetic Resonance (NMR) spectroscopy into a composite score that captures multi-dimensional metabolic perturbations, thereby offering richer insight than any single marker alone (15). This index consists of six key constituents: citrate, small HDL particles, acetylated glycoproteins (GlycA), plus the branched-chain amino acids (BCAAs) triad isoleucine, leucine and valine. Generally, small HDL particles and GlycA are utilized to capture and reflect the degree of systemic inflammation in the body, whereas citrate and the remaining three amino acids are employed to assess metabolic malnutrition, with the calculation results represented by the inflammation vulnerability index (IVX) and metabolic malnutrition index (MMX), respectively (16, 17). MVX is a composite score based on six biomarkers.

The clinical pertinence of the MVX has been tentatively corroborated by previous studies, which demonstrate a significant correlation between elevated MVX levels and all-cause mortality (18), with underlying pathophysiology potentially implicating endoplasmic reticulum stress (ERS) and oxidative insult (19). The metabolic footprints captured by MVX are thought to surface during the preclinical window, the silent prodromal phase (20), suggesting that MVX may hold unique value for SLE prediction and disease risk stratification that differs from traditional clinical indicators. Actually, substantial evidence has reported the association between inflammation and SLE (21–23). However, whether MVX, which integrates metabolic factors, demonstrates a stronger association with SLE than the inflammation-only index IVX remains unexplored. Additionally, systematic research exploring the relationship between MVX (along with its sub-indices IVX and MMX) and SLE development is lacking. Therefore, this study aims to estimate the association between MVX and SLE using a large-scale prospective independent clinical cohort.

## 2 Materials and methods

### 2.1 UK Biobank study design

**Figure 1:**
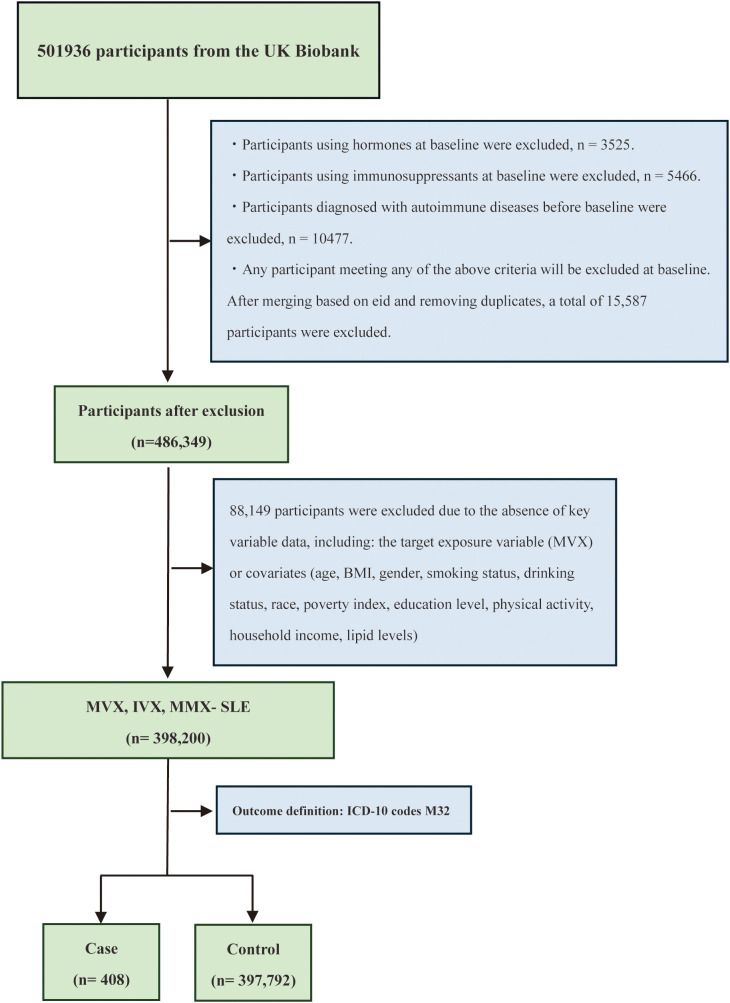
Flowchart of participant selection for analysis of MVX, IVX, MMX and SLE from the UK Biobank. This flowchart illustrates the process of selecting participants from the UK Biobank for a study examining the relationship between MVX, IVX, MMX and SLE. Initially, 501,936 participants were included. Participants using hormones or immunosuppressants at baseline, those diagnosed with autoimmune diseases before baseline, and any participant meeting these criteria were excluded, resulting in the removal of 15,587 participants. After merging based on unique identifiers (eid) and removing duplicates, 486,349 participants remained. Further exclusions due to missing key variable data reduced the sample size to 398,200 for MVX, IVX, MMX-SLE analysis. The outcome definition was based on ICD-10 codes M32, leading to 408 cases and 397,792 controls.

This investigation harnessed data from the UKB, comprising a cohort of more than 500,000 native UK residents recruited between 2006 and 2010. Extensive baseline data were systematically collected, covering sociodemographic characteristics, physical measurements, lifestyle factors, medication history, clinical history, and laboratory biochemical indicators. Participants were sequentially excluded if they had baseline glucocorticoid use (n=3,525), baseline immunosuppressant use (n=5,466), or pre-existing autoimmune diseases (n=10,477). These exclusions were implemented to minimize confounding by medication and avoid reverse causality, as autoimmune conditions and their treatments (e.g., glucocorticoids) significantly alter metabolic profiles and may precede SLE onset. Furthermore, individuals with missing covariate data (n=88,149) were excluded to ensure the robustness of the primary analysis, resulting in a final analytic cohort of 398,200 participants. **Figure 1** illustrates the detailed participant screening process.

To address the potential impact of missing data, we additionally performed a sensitivity analysis using multiple imputation by chained equations (MICE) (24) to create a complete dataset (N = 461,316), as detailed in the statistical analysis section. **Figure 1** illustrates the detailed participant screening process. This study was supported by UKB project application number 578405. The study was conducted using the UKB resources, which operate under ethical clearance granted by the North West Multi-center Research Ethics Committee.

### 2.2 Ascertainment of outcomes

Using field ID p20003, baseline glucocorticoid use was defined to include prednisolone (4 coding events), dexamethasone, prednisone, hydrocortisone (1 coding event each), and methylprednisolone (2 coding events), with use restricted to oral preparations to exclude ophthalmic solutions or topical creams. Immunosuppressant use was also assessed via field ID p20003, covering cyclophosphamide, mycophenolate, leflunomide, ciclosporin, tacrolimus, hydroxychloroquine, sulfasalazine, methotrexate, and azathioprine (See **Supplementary Methods** for the 16 event codes). The baseline exclusion criteria encompassed any pre-existing autoimmune disease defined by the International Classification of Diseases, 10th Revision (ICD-10) codes, specifically including inflammatory bowel disease (K50, K51), rheumatoid arthritis (M05, M06, M08.0), ankylosing spondylitis (M45, M081), type 1 diabetes mellitus (E10), systemic sclerosis (M34), sicca syndrome (M350), thyrotoxicosis with diffuse goiter (E050), autoimmune thyroiditis (E063), polyarteritis nodosa (M30), dermatomyositis (M33), and psoriasis (L40). The exposure variables MVX, IVX, and MMX were evaluated during the UKB baseline assessment. The study outcome was defined as the occurrence of SLE, identified by the ICD-10 code M32. Follow-up commenced at the baseline visit and ended at the earliest of the following events: diagnosis of the outcome, death, loss to follow-up, or March 31, 2023.

### 2.3 Metabolic biomarker profiling and MVX calculation

Metabolic biomarker profiles were obtained from EDTA plasma samples collected at the UKB baseline assessment. Nightingale Health performed high-throughput NMR metabolic profiling on theses randomly selected samples. comprehensive information regarding the experimental and quality control protocols have been previously described (25). The sex-specific calculation followed previously established methods (15). MVX was constructed based on six specific metabolites: citrate, isoleucine, glycA, leucine, valine, and small HDL particles. The MVX calculation comprises a three-step algorithm: initially, glycA and small HDL particles are used to determine the IVX; subsequently, the four remaining metabolites are used to calculate the MMX; ultimately, IVX and MMX are aggregated via specific coefficients to yield the final MVX score. MVX scores (range: 1–100) are positively correlated with metabolic fragility, with lower scores suggesting better health and higher scores suggest worse metabolic vulnerability. The specific formula is provided in the **Supplementary Methods**.

### 2.4 Ascertainment of covariates

The independent associations of MVX, IVX and MMX with SLE were evaluated separately. To ensure the robustness of the results, a series of potential confounding factors were corrected in the analysis. The covariates encompassed demographic factors including age, sex, and ethnicity, as well as socioeconomic indicators. This category included educational attainment, household income, physical activity (26), and the Townsend Deprivation Index (TDI). Specifically, education was trichotomized as unknown, college or university degree, or other (27). Meanwhile, household income was stratified into five tiers ranging from less than 18,000 to greater than 100,000 (28). Lifestyle factors smoking and drinking status were categorized as current, never, or previous (29). Anthropometric measures included BMI, classified as obese (BMI ≥30.0 kg/m²), overweight (BMI between 25.0–29.9 kg/m²) and normal (BMI between 18.5–24.9 kg/m²) (30). Furthermore, adjustments were made for biochemical markers, including triglycerides, and cholesterol. Clinical history adjusted for cancer history, cardiovascular disease (CVD) history, chronic liver disease (CLD) history, and type 2 diabetes (T2DM) history (ICD-10 E11). (Study variables and UKB field IDs are listed in the **Supplementary Methods**).

### 2.5 Statistical analysis

Data extraction, management, and preprocessing were conducted using the FastUKB 3.0 toolkit (v3.0) within the R 3.6.3 environment (31). Unlike the standard UK Biobank research analysis platform (RAP) interface, which relies on custom structured query language coding and restricts variable selection, FastUKB utilizes a graphical user interface to support batch extraction of unlimited variables across multiple domains. Beyond retrieval, the tool implements an automated medical data quality control framework that handles specific missing codes, detects physiologically implausible outliers, performs logical cross-validation, and standardizes variable encodings to ensure data integrity.

Based on the normality test outcomes: continuous data with a normal distribution are summarized as mean ± SD and compared via Student’s t-test or ANOVA; while non-normally distributed metrics are reported as median (IQR) and subjected to the Krusal–Wallis procedures or Mann–Whitney U. Regarding categorical variables, we calculated proportions and used the chi-square test to check for intergroup variations. Statistical significance was established at P < 0.05 (two-tailed). We examined the proportionality of hazards via Schoenfeld residuals. Subsequently, the association between MVX (IVX, MMX) and SLE, was quantified using multivariate Cox models to calculate hazard ratios (HRs) accompanied by 95% confidence intervals (CI). model 1 was adjusted for ethnicity, gender and age. Building upon model 1, model 2 additionally adjusted for drinking status, smoking status, TDI, BMI, household income, education level, physical activity. model 3 comprised all variables in Models 1 and 2, plus adjustments for cancer, CVD history, and CLD history, T2DM history, cholesterol, triglycerides. Finally, restricted cubic splines (RCS) were employed to evaluate potential non-linear trends between exposures and SLE risks. Four knots were placed at the 5th, 35th, 65th, and 95th percentiles of the distribution for MVX, IVX, and MMX, respectively, in accordance with Harrell’s recommendations for survival models. Subgroup analysis was performed based on gender, BMI, smoking and alcohol consumption, and physical activity status to explore the associations between the exposure factors and SLE across different categories. Potential multicollinearity between MVX and SLE in the adjusted models was assessed using the generalized variance inflation factor (GVIF) calculated with the car package.

To further verify the robustness of the study findings, we employed a three-year lag exclusion strategy in sensitivity analyses to mitigate bias from prevalent undiagnosed cases and reverse causation. Furthermore, based on the fully adjusted model, C-reactive protein was additionally included as a covariate for re-fitting. This was done to incorporate the inflammatory indicator into the regression equation and control for its potential confounding effect on the association between the exposure variable and the outcome. To verify robustness against violations of the proportional hazards assumption, we employed extended Cox models with time-dependent covariates and stratification for sensitivity analysis. For exposures and SLE, we progressively introduced time-dependent terms and applied stratification. In models 1 and 2, we included age as a time-dependent covariate and stratified by gender. In model 3, we included age as a time-dependent covariate and stratified by both gender and CVD history. All other covariates included in the three models remained unmodified (meeting the proportional hazards assumption). Finally, to address the potential impact of missing data, we conducted a sensitivity analysis using a dataset constructed via multiple imputation. Missing covariates were imputed using the MICE (24) package in R. Predictive mean matching was employed for continuous variables, while polytomous regression was used for binary and unordered categorical variables. The imputation procedure was configured to generate 5 complete datasets (m = 5) with the number of iterations set to 10 (maxit = 10). Parameter estimates and standard errors were pooled according to Rubin’s rules (32).

Given that the MVX was originally constructed to capture cardiovascular mortality risk and has been strongly associated with all-cause mortality in prior studies (15, 20), individuals with high MVX scores face a substantial competing risk of death before the onset of SLE. To account for this potential bias and provide a more accurate estimation of the real-world risk, we employed Fine-Gray subdistribution hazard models. In this analysis, all-cause mortality and CVD-specific mortality were treated as competing events. The models were fitted using the crr function from the cmprsk package in R (33). This approach allows us to assess the net cumulative incidence of SLE in the context of high metabolic vulnerability.

### 2.6 Sensitivity analysis of metabolite contributions

To identify the specific metabolites driving the association between the MVX score and SLE risk, we performed a sensitivity analysis using a leave-one-out approach. Briefly, we recalculated the MVX score by sequentially excluding one metabolite at a time (replacing the excluded variable with the population mean) while retaining the remaining metabolites in the score. We then re-evaluated the association between the revised MVX score and SLE risk using Cox proportional hazards models.

## 3 Results

### 3.1 Baseline characteristics

A total of 398,200 participants were included in the final analysis and were categorized into three groups based on the tertiles (T1, T2, T3) of the MVX score. The baseline characteristics of the study population stratified by MVX levels are presented in **Table 1**. Participants in the highest MVX tertile (T3) were significantly older, with a median age of 59.0 years, compared to those in the lowest tertile (T1, 57.0 years) (P < 0.01). The gender distribution varied notably across MVX categories; the proportion of females increased progressively across MVX tertiles, from 20.74% in T1 to 79.59% in T3 (P<0.001). While the majority of participants were White across all groups, the proportion of Non-White individuals was lowest in T2 (4.93%) and slightly higher in T1 and T3 (P<0.01). Regarding lifestyle factors, significant trends were observed for alcohol consumption and smoking status. Specifically, the prevalence of current drinkers decreased from T1 to T3 (93.80% vs. 89.44%), whereas current smoking increased from 9.88% in T1 to 12.75% in T3 (P<0.01 for both). Physical activity levels showed a graded decrease, with 66.45% of participants in T1 reporting engagement in physical activity compared to only 56.81% in T3 (P<0.01). In terms of metabolic and clinical health parameters, higher MVX scores were associated with adverse metabolic profiles. The median BMI and levels of triglycerides and cholesterol were significantly higher in the T3 group compared to the T1 and T2 groups (all P<0.01). History of chronic diseases also differed significantly by MVX category. The prevalence of Cancer and CVD history was lowest in the T1 group and highest in the T3 group. For instance, the history of CVD was 27.66% in T1 and rose to 33.00% in T3 (P<0.01). In summary, significant elevations in MVX levels were observed in populations characterized by older age, female sex, and a higher burden of metabolic and cardiovascular adverse profiles.

**Table 1:** Baseline characteristics of participants according to MVX tertiles.

Characteristic	T1	T2	T3	p-value_2_
	N = 133,2091	N = 132,7671	N = 132,2241	
Age				<0.001
Median (Q1, Q3)	57.00 (49.00, 63.00)	57.00 (50.00, 63.00)	59.00 (51.00, 64.00)	
Gender				<0.001
Female	27,634 (20.74%)	81,608 (61.48%)	105,242 (79.59%)	
Male	105,575 (79.26%)	51,149 (38.52%)	26,982 (20.41%)	
Ethnicity				<0.001
Non White	7,662 (5.75%)	6,542 (4.93%)	6,818 (5.16%)	
White	125,547 (94.15%)	126,255 (95.07%)	125,406 (94.74%)	
Drinking status				<0.001
Current	124,948 (93.80%)	123,563 (93.07%)	118,263 (89.44%)	
Never	3,899 (2.93%)	5,129 (3.86%)	8,201 (6.20%)	
Previous	4,362 (3.27%)	4,075 (3.07%)	5,760 (4.36%)	
Smoking status				<0.001
Current	13,162 (9.88%)	13,630 (10.27%)	16,862 (12.75%)	
Never	71,906 (53.80%)	73,088 (55.05%)	71,457 (54.04%)	
Previous	48,141 (36.32%)	46,049 (34.68%)	43,905 (33.21%)	
TDI				<0.001
Median (Q1, Q3)	-2.27 (-3.72, 0.27)	-2.22 (-3.69, 0.35)	-1.99 (-3.55, 0.83)	
BMI				<0.001
Normal	47,141 (35.39%)	46,164 (34.77%)	35,886 (27.14%)	
Obese	24,930 (18.71%)	29,796 (22.44%)	42,514 (32.15%)	
Overweight	60,538 (45.45%)	56,135 (42.28%)	53,265 (40.29%)	
Underweight	600 (0.45%)	672 (0.51%)	559 (0.42%)	
Education				<0.001
College	50,898 (38.21%)	43,296 (32.61%)	34,290 (25.93%)	
Other	61,997 (46.54%)	66,819 (50.33%)	68,299 (51.65%)	
UnKnown	20,314 (15.25%)	22,652 (17.06%)	29,635 (22.42%)	
Physical activity				<0.001
Non	44,691 (33.55%)	50,566 (38.09%)	57,106 (43.19%)	
Yes	88,518 (66.45%)	82,201 (61.91%)	75,118 (56.81%)	
Cancer				<0.001
No	124,656 (93.58%)	121,905 (91.82%)	119,273 (90.21%)	
Yes	8,553 (6.42%)	10,862 (8.18%)	12,951 (9.79%)	
CVD history				<0.001
No	96,367 (72.34%)	96,122 (72.40%)	88,592 (67.00%)	
Yes	36,842 (27.66%)	36,645 (27.60%)	43,632 (33.00%)	
CLD history				<0.001
No	132,144 (99.20%)	132,061 (99.47%)	131,468 (99.43%)	
Yes	1,065 (0.80%)	706 (0.53%)	756 (0.57%)	
T2DM history				<0.001
No	130,661 (98.09%)	130,769 (98.50%)	129,765 (98.14%)	
Yes	2,548 (1.91%)	1,998 (1.50%)	2,459 (1.86%)	
Cholesterol				<0.001
Median (Q1, Q3)	5.33 (4.63, 6.04)	5.72 (5.01, 6.46)	5.94 (5.16, 6.76)	
Triglycerides				<0.001
Median (Q1, Q3)	1.31 (0.96, 1.82)	1.45 (1.03, 2.07)	1.77 (1.21, 2.58)	

Data are presented as median (interquartile range) or number (percentage).p values were calculated using the Kruskal-Wallis rank sum test for continuous variables and Pearson’s Chi-squared test for categorical variables. Participants were divided into three groups (T1, T2, T3) by MVX levels (range: T1 lowest, T3 highest). MVX, metabolic vulnerability index. ^1^n (%) ^2^Kruskal-Wallis rank sum test; Pearson's Chi-squared test.

### 3.2 Cox proportional hazards model results

Multicollinearity diagnostics revealed no significant multicollinearity in this study dataset (see **Supplementary File**). **Table 2** presents the Cox proportional hazards model results for MVX, IVX, and MMX regarding the risk of SLE. As the covariates violated the proportional hazards assumption, the results reflect the average hazard intensity over the 13-year follow-up (20). For MVX (**Table 2A**), in the fully adjusted Model 3, every one-standard-deviation (per SD) increase in MVX was associated with a 44% increased risk of SLE (HR = 1.44, 95% CI: 1.28-1.62, P<0.01). Tertile-based analysis revealed that participants in the highest tertile (T3) faced an 89% higher risk of SLE relative to the reference group T1 (HR = 1.89, 95% CI: 1.35-2.63, P<0.01). Regarding IVX (**Table 2B**), in the fully adjusted Model 3, every one-standard-deviation (per SD) increase in IVX was associated with a 34% increased risk of SLE (HR = 1.34, 95% CI: 1.17-1.54, P<0.01). Participants in the highest tertile (T3) had an 85% higher risk of SLE compared to T1 (HR = 1.85, 95% CI: 1.25-2.74, P<0.01). In the case of MMX (**Table 2C**), in the fully adjusted Model 3, every one-standard-deviation (per SD) increase in MMX was associated with a 24% increased risk of SLE (HR = 1.24, 95% CI: 1.14-1.36, P<0.01). Tertile comparisons (T3 vs. T1) yielded a hazard ratio of 1.82 (95% CI: 1.42-2.33, P<0.01). Detailed results for all models are shown in **Table 2**. Irrespective of the adjustment for covariates, the association strength of the composite index MVX for SLE was superior to that of the single indices IVX or MMX, while the association strength of IVX was superior to that of MMX in Model 3 per SD comparison.

**Table 2:** Relationship between exposures and SLE risk based on the cox proportional hazards model.

A: Cox proportional hazards results for MVX on SLE											
Variable	Case N	Control N	Model 1			Model 2			Model 3		
			HR	95% CI	P value	HR	95% CI	P value	HR	95% CI	P value
MVX (per SD)	408	397,792	1.41	1.27-1.56	<0.01	1.46	1.30-1.64	<0.01	1.44	1.28-1.62	<0.01
MVX T1	65	133,144	1.00	1.00 (Ref)	1.00	1.00	1.00 (Ref)	1.00	1.00	1.00 (Ref)	1.00
MVX T2	116	132,651	1.11	0.81-1.54	0.51	1.16	0.83-1.62	0.38	1.16	0.84-1.62	0.36
MVX T3	227	131,997	1.84	1.35-2.51	<0.01	1.88	1.35-2.63	<0.01	1.89	1.35-2.63	<0.01
B: Cox proportional hazards results for IVX on SLE											
	Case N	Control N	Model 1			Model 2			Model 3		
			HR	95% CI	Pvalue	HR	95% CI	Pvalue	HR	95% CI	Pvalue
IVX (per SD)	408	397,792	1.29	1.15-1.45	<0.01	1.37	1.20-1.58	<0.01	1.34	1.17-1.54	<0.01
IVX T1	60	133,449	1.00	1.00 (Ref)	1.00	1.00	1.00 (Ref)	1.00	1.00	1.00 (Ref)	1.00
IVX T2	117	132,256	1.05	0.73-1.50	0.78	1.13	0.78-1.64	0.50	1.14	0.79-1.65	0.49
IVX T3	231	132,087	1.68	1.18-2.39	<0.01	1.85	1.25-2.73	<0.01	1.85	1.25-2.74	<0.01
C: Cox proportional hazards results for MMX on SLE											
	Case N	Control N	Model 1			Model 2			Model 3		
			HR	95% CI	Pvalue	HR	95% CI	Pvalue	HR	95% CI	Pvalue
MMX (per SD)	408	397,792	1.24	1.14-1.35	<0.01	1.24	1.13-1.36	<0.01	1.24	1.14-1.36	<0.01
MMX T1	168	131,734	1.00	1.00 (Ref)	1.00	1.00	1.00 (Ref)	1.00	1.00	1.00 (Ref)	1.00
MMX T2	101	133,495	1.25	0.96-1.62	0.09	1.30	1.00-1.70	>0.05	1.30	1.01-1.69	0.04
MMX T3	139	132,563	1.80	1.42-2.29	<0.01	1.82	1.42-2.33	<0.01	1.82	1.42-2.33	<0.01

MVX, IVX, and MMX were categorized into tertiles (T1–T3), with the lowest tertile (T1) designated as the reference group. HRs and corresponding 95% CIs were derived using Cox proportional hazards regression models. Three progressively adjusted models were constructed: Model 1 was adjusted for age, gender, and ethnicity. Model 2 was further adjusted for drinking status, smoking status, TDI, BMI, household income, education, and physical activity. Model 3 was additionally adjusted for cancer history, T2DM history, history of cardiovascular disease, history of chronic liver disease, and lipid levels. All covariates were assessed at baseline. P-values were two-sided, with statistical significance defined as P < 0.05.

HR, Hazard ratio; CI, Confidence interval; TDI, Townsend Deprivation Index; Ref, Reference group; MVX, Metabolic Vulnerability Index; IVX, Inflammation Vulnerability Index; MMX, Metabolic Malnutrition Index.

### 3.3 Subgroup analysis of SLE

Subgroup analyses based on the fully adjusted Model 3 demonstrated consistent positive associations between the MVX and SLE risk across various strata (**Table 3**). Regarding BMI categories, MVX remained significantly associated with SLE in the normal weight (HR = 1.55, 95% CI: 1.27-1.87, P<0.01), overweight (HR = 1.31, 95% CI: 1.08-1.60, P<0.01), and obese (HR = 1.37, 95% CI: 1.09-1.72, P<0.01) groups. In terms of gender, while a significant association was observed in females (HR = 1.59, 95% CI: 1.39-1.81, P<0.01), the association was not significant in males (HR = 0.98, 95% CI: 0.81-1.19, P>0.05). Furthermore, MVX showed significant predictive value in both the smoker (HR = 1.67, 95% CI: 1.40-1.99, P<0.01) and never-smoker (HR = 1.24, 95% CI: 1.04-1.50, P = 0.01) groups. Similarly, for physical activity, significant associations persisted in both the “Yes” (HR = 1.51, 95% CI: 1.28-1.77, P<0.01) and “No” (HR = 1.38, 95% CI: 1.16-1.66, P<0.01) groups, as well as in the “Yes” drinking (HR = 1.46, 95% CI: 1.29-1.66, P<0.01) and “Never” drinking (HR = 1.64, 95% CI: 1.09-2.46, P = 0.01) groups. Collectively, these findings underscore the robustness of MVX as a risk factor for SLE across diverse demographic and lifestyle profiles.

**Table 3:** Subgroup analysis.

A: Subgroup analyses of the association between exposures and SLE stratified by BMI in the fully adjusted model 3									
Variable	BMI normal (case=148, control=128,283)			BMI overweight (case=148, control=169,368)			BMI obese (case=112, control=100,141)		
	HR	95% CI	P value	HR	95% CI	P value	HR	95% CI	P value
MVX (per SD)	1.55	1.27-1.87	<0.01	1.31	1.08-1.60	<0.01	1.37	1.09-1.72	<0.01
IVX (per SD)	1.35	1.19-1.54	<0.01	1.18	0.93-1.49	0.56	1.26	0.97-1.64	0.08
MMX (per SD)	1.21	1.05-1.39	<0.01	1.28	1.09-1.50	<0.01	1.28	1.06-1.55	0.01
B: Subgroup analyses of the association between exposures and SLE stratified by gender in the fully adjusted model 3									
				Female (case=336, control=213,986)			Male (case=72, control=183,806)		
				HR	95% CI	Pvalue	HR	95% CI	Pvalue
MVX (per SD)				1.59	1.39-1.81	<0.01	0.98	0.81-1.19	<0.01
IVX (per SD)				1.53	1.31-1.79	1.00	0.92	0.76-1.11	0.37
MMX (per SD)				1.21	1.10-1.33	<0.01	1.80	1.19-2.70	<0.01
C: Subgroup analyses of the association between exposures and SLE stratified by smoking in the fully adjusted model 3									
				Yes (case=218, control=181,654)			Never (case=190, control=216,138)		
				HR	95% CI	Pvalue	HR	95% CI	Pvalue
MVX (per SD)				1.67	1.40-1.99	<0.01	1.24	1.04-1.50	0.01
IVX (per SD)				1.54	1.25-1.89	<0.01	1.16	0.93-1.44	0.12
MMX (per SD)				1.33	1.16-1.53	<0.01	1.18	1.03-1.35	0.01
D: Subgroup analyses of the association between exposures and SLE stratified by physical activity in the fully adjusted model 3									
				Yes (case=234, control=240,649)			No (case=174, control=157,143)		
				HR	95% CI	Pvalue	HR	95% CI	Pvalue
MVX (per SD)				1.51	1.28-1.77	<0.01	1.38	1.16-1.66	<0.01
IVX (per SD)				1.44	1.19-1.74	<0.01	1.31	1.06-1.61	0.01
MMX (per SD)				1.24	1.09-1.40	<0.01	1.20	1.04-1.38	0.01
E: Subgroup analyses of the association between exposures and SLE stratified by drinking in the fully adjusted model 3									
				Yes (case=371, control=379,702)			Never (case=37, control=18,090)		
				HR	95% CI	Pvalue	HR	95% CI	Pvalue
MVX (per SD)				1.46	1.29-1.66	<0.01	1.64	1.09-2.46	0.01
IVX (per SD)				1.39	1.20-1.62	<0.01	1.49	0.93-2.39	0.09
MMX (per SD)				1.23	1.12-1.37	<0.01	1.30	0.97-1.76	0.08

Model 1 was adjusted for age, gender, and ethnicity. Model 2 was further adjusted for drinking status, smoking status, TDI, BMI, household income, education, and physical activity. Model 3 was additionally adjusted for cancer history, T2DM history, history of cardiovascular disease, history of chronic liver disease, and lipid levels. All covariates were assessed at baseline. P-values were two-sided, with statistical significance defined as P < 0.05.

HR, Hazard ratio; CI, Confidence interval; TDI, Townsend Deprivation Index; Ref, Reference group; MVX, Metabolic Vulnerability Index; IVX, Inflammation Vulnerability Index; MMX, Metabolic Malnutrition Index.

### 3.4 RCS analysis results

**Figure 2:**
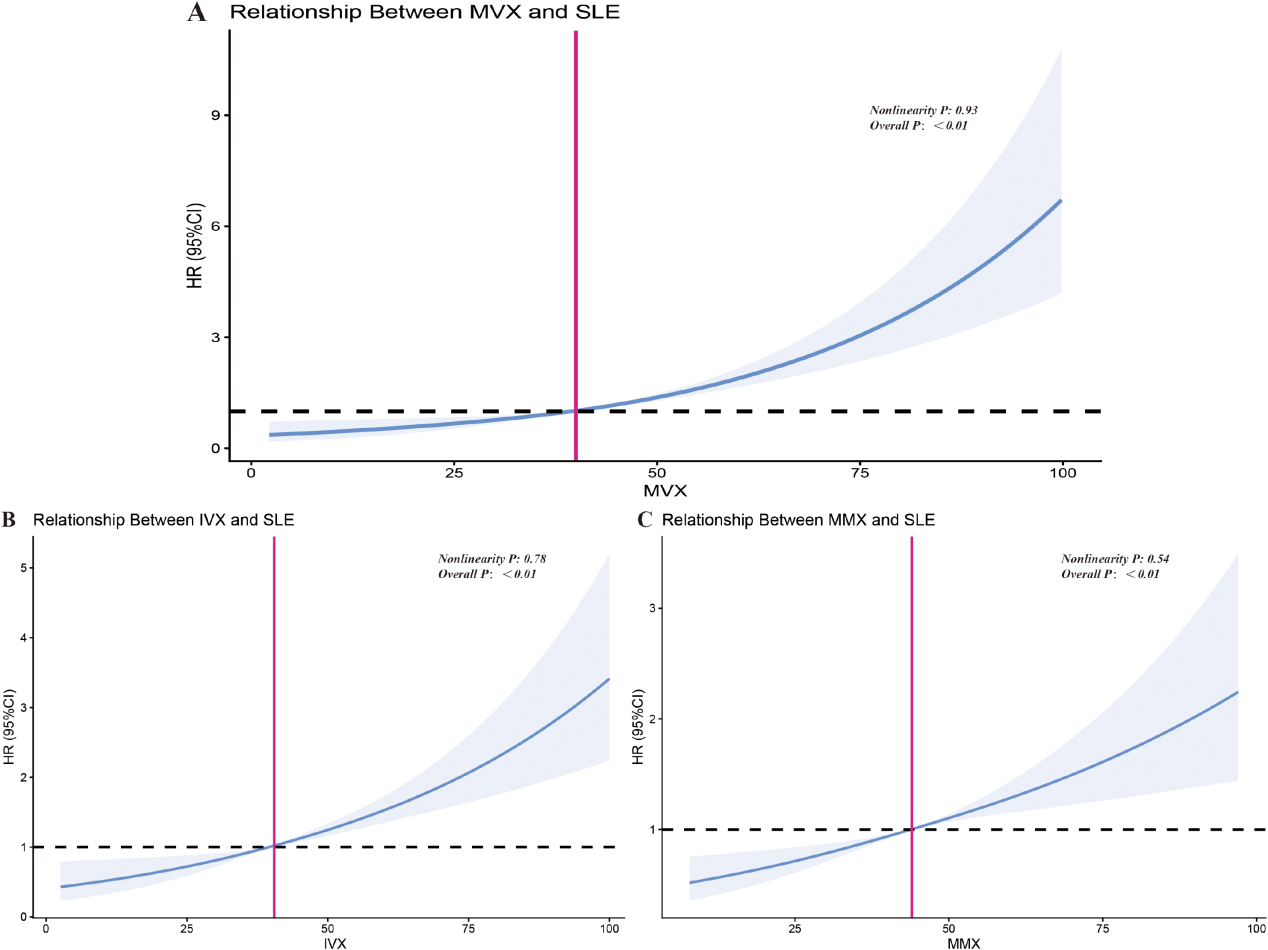
Restricted cubic spline analysis of the relationship between MVX, IVX, MMX and SLE. **(A–C)** depict the dose-response relationships between MVX **(A)**, IVX **(B)**, and MMX **(C)** and the risk of SLE. The blue solid lines represent the estimated hazard ratios (HRs), with the 95% confidence intervals (CIs) shaded in gray. The dashed black line indicates an HR of 1 (reference). The pink vertical lines mark the thresholds at which the risk of SLE begins to increase (MVX: 36, IVX: 40, MMX: 45). Collectively, the spline curves illustrate a linear dose-response relationship, where the risk of SLE rises as the index increases beyond these identified thresholds.

RCS analysis revealed linear relationships between MVX, IVX, and MMX with SLE (**Figures 2A–C**). Elevated levels of all three indices were significantly associated with the risk of SLE and exhibited a clear dose-response relationship, in which the risk of SLE gradually increased as the index levels rose. The p-values for nonlinearity were not significant (MVX: P = 0.93, IVX: P = 0.78, MMX: P = 0.54), suggesting that the relationships between MVX, IVX, and MMX with SLE are linear. In the RCS plots, the blue lines represent the HRs with their corresponding 95% CIs, showing a consistent upward trend across the range of each index. There is no evidence of inflection points or changes in the slope of the curves, supporting the linearity of these associations. The red lines in the plots represent the risk thresholds. When MVX exceeds 36, IVX exceeds 40, and MMX exceeds 45, the risk of SLE increases as the indices rise. Among the three indices, MVX had the most significant impact on SLE, as it showed the greatest increase in HR across its range.

### 3.5 Sensitivity analysis results

In the sensitivity analyses, we employed four strategies to verify the robustness of the observed associations: a 3-year lag exclusion, additional adjustment for CRP, extended Cox models with time-dependent covariates, and multiple imputation for missing data. The results are summarized in **Table 4**. For the 3-year exclusion analysis (**Table 4A**), where participants with follow-up less than three years were excluded, the significant associations persisted across all models. Specifically, the HRs for MVX ranged from 1.39 to 1.44 (model 3 HR = 1.42, 95% CI: 1.25-1.62). Similar robust associations were observed for IVX and MMX. In the CRP adjustment analysis (**Table 4B**), after further controlling for CRP in the fully adjusted model, the association between MVX and SLE risk remained significant (HR = 1.38, 95% CI: 1.22-1.57). To address violations of the proportional hazards assumption, we utilized extended Cox models with time-dependent covariates and stratification (**Table 4C**). The results confirmed the stability of the primary findings, with MVX showing a consistent hazard ratio of 1.48 (95% CI: 1.34-1.62) in the stratified model 3. Finally, the multiple imputation analysis (**Table 4D**), which addressed missing data bias by enlarging the sample to 461,316 participants, yielded comparable results. In the imputed dataset, MVX remained strongly associated with SLE risk (HR = 1.46, 95% CI: 1.31-1.63), and the relationships for IVX and MMX remained robust. Additionally, landmark analyses at 5 and 10 years were performed (**Tables 4E, F**). While very mild regression dilution was observed, the key finding is that these associations remained statistically significant across all timeframes, underscoring the robustness of our results. Collectively, these sensitivity analyses confirm that the associations between metabolic vulnerability indices and SLE risk are stable and not significantly influenced by reverse causality, inflammatory status, proportional hazards violations, or missing data. Baseline characteristics comparing the imputed data and data with missing covariates are shown in the supplemental results.

**Table 4:** Sensitivity analysis: relationship between exposures and SLE risk based on the cox proportional hazards models.

A: Sensitivity analysis (3-year exclusion) of exposures on SLE											
Variable	Case N	Control N	Model 1			Model 2			Model 3		
			HR	95% CI	P value	HR	95% CI	P value	HR	95% CI	P value
MVX (per SD)	333	394,146	1.39	1.24-1.55	<0.01	1.44	1.26-1.64	<0.01	1.42	1.25-1.62	<0.01
IVX (per SD)	333	394,146	1.26	1.11-1.43	<0.01	1.33	1.14-1.55	<0.01	1.30	1.12-1.52	<0.01
MMX (per SD)	333	394,146	1.25	1.13-1.38	<0.01	1.26	1.14-1.40	<0.01	1.27	1.14-1.40	<0.01
B: Sensitivity analysis (plus CRP adjustment) of exposures on SLE											
	Case N	Control N	Model 1			Model 2			Model 3 + CRP		
			HR	95% CI	Pvalue	HR	95% CI	Pvalue	HR	95% CI	Pvalue
MVX (per SD)	408	397,792	1.41	1.27-1.57	<0.01	1.47	1.30-1.65	<0.01	1.38	1.22-1.57	<0.01
IVX (per SD)	408	397,792	1.29	1.16-1.50	<0.01	1.38	1.20-1.58	<0.01	1.26	1.08-1.46	<0.01
MMX (per SD)	408	397,792	1.24	1.14-1.35	<0.01	1.24	1.14-1.36	<0.01	1.24	1.13-1.36	<0.01
C: Sensitivity analysis using time-dependent covariates and stratification: association between exposures and SLE											
	Case N	Control N	Model 1			Model 2			Model 3		
			HR	95% CI	Pvalue	HR	95% CI	Pvalue	HR	95% CI	Pvalue
MVX (per SD)	408	397,792	1.45	1.34-1.58	<0.01	1.50	1.37-1.66	<0.01	1.48	1.34-1.62	<0.01
IVX (per SD)	408	397,792	1.30	1.19-1.42	<0.01	1.40	1.26-1.57	<0.01	1.35	1.21-1.51	<0.01
MMX (per SD)	408	397,792	1.28	1.19-1.37	<0.01	1.28	1.19-1.37	<0.01	1.30	1.21-1.39	<0.01
D: Sensitivity analysis: association between exposures and SLE based on multiple imputation											
	Case N	Control N	Model 1			Model 2			Model 3		
			HR	95% CI	Pvalue	HR	95% CI	Pvalue	HR	95% CI	Pvalue
MVX (per SD)	481	460,835	1.42	1.29-1.56	<0.01	1.48	1.33-1.65	<0.01	1.46	1.31-1.63	<0.01
IVX (per SD)	481	460,835	1.29	1.17-1.43	<0.01	1.39	1.23-1.58	<0.01	1.34	1.18-1.53	<0.01
MMX (per SD)	481	460,835	1.24	1.15-1.35	<0.01	1.24	1.14-1.35	<0.01	1.24	1.14-1.35	<0.01
E: Sensitivity analysis with a 5-year landmark assumption											
	Case N	Control N	Model 1			Model 2			Model 3		
			HR	95% CI	Pvalue	HR	95% CI	Pvalue	HR	95% CI	Pvalue
MVX (per SD)	126	398,074	1.54	1.28-1.85	<0.01	1.56	1.27-1.92	<0.01	1.50	1.22-1.86	<0.01
IVX (per SD)	126	398,074	1.40	1.14-1.70	<0.01	1.47	1.16-1.88	<0.01	1.42	1.11-1.82	<0.01
MMX (per SD)	126	398,074	1.26	1.08-1.47	<0.01	1.24	1.06-1.46	<0.01	1.25	1.07-1.46	<0.01
F: Sensitivity analysis with a 10-year landmark assumption											
	Case N	Control N	Model 1			Model 2			Model 3		
			HR	95% CI	Pvalue	HR	95% CI	Pvalue	HR	95% CI	Pvalue
MVX (per SD)	279	397,921	1.52	1.34-1.72	<0.01	1.55	1.35-1.79	<0.01	1.50	1.31-1.73	<0.01
IVX (per SD)	279	397,921	1.38	1.21-1.58	<0.01	1.43	1.21-1.68	<0.01	1.38	1.17-1.63	<0.01
MMX (per SD)	279	397,921	1.26	1.14-1.40	<0.01	1.28	1.15-1.43	<0.01	1.28	1.16-1.43	<0.01

Model 1 was adjusted for age, gender, and ethnicity. Model 2 was further adjusted for drinking status, smoking status, TDI, BMI, household income, education, and physical activity. Model 3 was additionally adjusted for cancer history, T2DM history, history of cardiovascular disease, history of chronic liver disease, and lipid levels. All covariates were assessed at baseline. P-values were two-sided, with statistical significance defined as P < 0.05. HR, Hazard ratio; CI, Confidence interval; TDI, Townsend Deprivation Index; Ref, Reference group; MVX, Metabolic Vulnerability Index; IVX, Inflammation Vulnerability Index; MMX, Metabolic Malnutrition Index; SD, Standard Deviation; CRP, C-Reactive Protein.

### 3.6 Fine and Gray’s competing risk models results

Results from Fine and Gray’s competing risk models are shown in supplement results. In this study cohort, treating all-cause mortality and CVD-specific mortality as competing events, the association between MVX and SLE remained robust, with subdistribution hazard ratios (SHRs) of 1.48 (95% CI: 1.34–1.63, P < 0.01) and 1.51 (95% CI: 1.37–1.67, P < 0.01) per 1-SD increase in MVX, respectively.

### 3.7 Metabolite contributions to the MVX-SLE association

GlycA was identified as the primary driver. The exclusion of glycA resulted in a significant reduction in the hazard ratio (HR) for SLE. In the fully adjusted model (Model 3), the HR decreased from 1.44 (95% CI: 1.28-1.62) for the original MVX to 1.29 (95% CI: 1.17-1.42) after excluding glycA, indicating its substantial contribution to the overall risk score. Leucine also contributed significantly to the association, with the HR dropping to 1.33 (95% CI: 1.18-1.50) upon its exclusion. In contrast, the exclusion of other metabolites, such as citrate, resulted in minimal changes to the association estimates (HR remaining at 1.43), suggesting that they drove the clinical association less strongly in this cohort. Detailed data are provided in the **Supplementary Results**.

## 4 Discussion

SLE is a complex autoimmune disease with a multifactorial pathogenesis, in which the relevance of metabolic abnormalities to disease progression is gaining increasing recognition (34). Elucidating the unique metabolic-immune alterations in the context of SLE holds significant clinical importance. Metabolomics enables the systematic characterization of dynamic changes in endogenous metabolites, offering a robust approach for identifying metabolic dysregulation and discovering potential biomarkers for the early prediction and clinical management of complex diseases (35, 36). Leveraging the recent expansion of metabolomic resources in the UKB, this study is the first to systematically assess the associations between SLE and inflammatory susceptibility, metabolic malnutrition, and metabolic vulnerability. Results demonstrated that MVX was consistently and significantly associated with SLE risk across multivariable-adjusted models, as well as in stratified and sensitivity analyses. Moreover, the strength of this association persistently exceeded that observed for IVX or MMX alone. These findings suggest that the onset of SLE may not be attributable to isolated inflammatory or metabolic abnormalities, but rather arises from a comprehensive background characterized by coupled metabolic–inflammatory dysregulation.

GlycA is a marker indicative of chronic low-grade inflammation (37). In patients with SLE, glycA correlates significantly with CVD risk factors (38, 39), while also exhibiting a positive correlation with disease activity and the extent of renal injury (40). Small HDL particles represent a subclass of HDL, with a particle size range of 7.3–8.2 nm as measured by NMR spectroscopy (41, 42). Currently, reports regarding the association between small HDL and CVD are inconsistent. Specifically, while univariate analyses based on NMR measurements show a positive correlation between small HDL and CVD risk, assessments via ion mobility analysis reported a significant protective association with CVD (43). Notably, small HDL serves as a key acceptor for cholesterol efflux from macrophages in the arterial wall and possesses antioxidant and anti-inflammatory properties (44). Consequently, preserving its normal proteolipid conformation allows small HDL to exert numerous cardioprotective biological functions, particularly definite anti-atherogenic effects. However, under inflammatory conditions, small HDL undergo functional remodeling. This process is characterized by diminished antioxidant and anti-inflammatory capacities, and in some cases, participation in complement activation and vascular inflammation (45–47). In the distinct pathological context of SLE, the dysregulated inflammatory milieu may induce extensive alterations in small HDL. Quantitatively, small HDL levels are significantly reduced in SLE patients regardless of CVD status (48, 49). Moreover, these particles show a decreasing trend across all age stages in SLE, with levels inversely correlating with disease activity (39). Regarding functionality, available evidence suggests that HDL derived from SLE patients possesses enhanced pro-inflammatory properties relative to healthy individuals (50), with small HDL specifically associated with the activation of the complement system (51). In summary, current evidence suggests that the complex characteristics of small HDL in SLE are characterized by quantitative depletion accompanied by pathological functional remodeling. Integrated with the IVX results from the UKB prospective cohort in this study, it suggests that during the subclinical phase preceding clinical diagnosis, the body may already exist in a sustained pro-inflammatory state, and this underlying inflammatory susceptibility effectively serves as an early warning signal for the risk of developing SLE. This provides a new, cost-effective screening strategy based on routine blood profiles for identifying high-risk populations during the preclinical phase, and offers a potential objective tool for optimizing the early intervention and clinical management of SLE.

In addition to inflammation, metabolism plays a critical role in the immune dysregulation of SLE. Citrate acts as a key hub in energy metabolism and serves not only as a crucial biomarker for mitochondrial function, but its serum levels also reflect the cellular balance between energy supply and demand (52). The catabolism of BCAAs, (including isoleucine, leucine, and valine) occurs primarily within the mitochondria; consequently, dysregulated circulating levels of these amino acids are typically viewed as indicators of mitochondrial dysfunction and disordered energy metabolism (53). Although current evidence has suggested the potential roles of citrate and BCAAs in immune metabolism, the specific pathological characteristics and interactions of this metabolic axis within the complex network of SLE require further elucidation. Nevertheless, several lines of evidence support the significance of this metabolic axis. For example, leucine-rich alpha-2-glycoprotein has been observed to increase with SLE disease activity (54). Additionally, there is an overactive citric acid cycle and abnormal BCAA catabolism in SLE patients (55). In fact, aberrant BCAA metabolism and an overactive citric acid cycle also play a crucial regulatory role in the abnormal activation of T cells and the shift in immune homeostasis (56–58). Given the high metabolic demand of this aberrant immune activation, the consequent rapid depletion of metabolic substrates may act as a driving force for disease progression.

In this study, the MMX index was found to be significantly positively correlated with SLE risk in the UK Biobank cohort, a finding that may reflect metabolic characteristics of the preclinical phase of SLE. Prior metabolomic analyses have validated the pervasive amino acid depletion in SLE patients (59, 60), which may be associated with inflammatory consumption and energy disorders in SLE. Within the MMX algorithm, lower levels of leucine and valine within the normal female range correspond to a higher MMX, suggesting based on the results of this study that SLE may involve the consumption of these two amino acids to some extent even in the subclinical stage. Reports on citrate evidence in SLE show a complex trend. Early metabolomic research on SLE found that serum citrate levels were lower in SLE patients compared to healthy individuals (60), reflecting the depletion of energy metabolism for basic immunity in SLE. However, recent study indicates that citrate levels increase with SLE onset age and drug exposure, suggesting that the combined effects of steroid-associated metabolic abnormalities, such as gluconeogenesis and glycolysis, and aging drive citrate accumulation despite early pathological consumption (39). In this context, the impact of isoleucine carries potential yet complex implications. Compared with healthy individuals, isoleucine levels are generally decreased in patients with SLE (59–61). Interestingly, in cases of moderate to severe coronary artery calcification (CAC) within the SLE, isoleucine levels manifest a distinct elevation (62). Moreover, a negative correlation has been found to exist between SLE activity and CAC (62). Investigations conducted in non-SLE populations have previously confirmed that elevated isoleucine levels are closely linked to increased CVD risk. The pathophysiology involves several key mechanisms, including insulin resistance, mTOR pathway activation, and the inflammatory response coupled with adverse remodeling of vascular smooth muscle cells; concurrently, metabolic dysfunction and oxidative stress act as direct drivers of atherosclerosis (63, 64). SLE progression involves abnormal energy metabolism and mitochondrial dysfunction (65). On one hand, inflammation drives the consumption of BCAAs; on the other, metabolic dysregulation leads to the accumulation of circulating isoleucine. Drawing on these findings, the increase in isoleucine observed in the SLE with severe calcification may be primarily attributed to the accumulation of SLE-related metabolic toxic burden. Conversely, the reduction of isoleucine during active SLE phases likely reflects excessive consumption and substrate depletion under acute immune activation. In this study, the MMX results to some extent reflect the metabolic instability during the subclinical phase of SLE, and existing evidence supports the complex coexistence of metabolic substrate consumption and pathological accumulation in SLE. In spite of these insights, the precise mechanisms operating within SLE require further elucidation.

Notably, MVX, as a composite index integrating the two dimensions of inflammation and metabolism, demonstrated superior prognostic value compared to IVX or MMX alone. In Model 3, each one SD increase in MVX was associated with a 44% increased risk of SLE; compared to the lowest tertile, participants in the highest tertile had an 89% increased risk of SLE. This finding further supports that the onset of SLE is not driven by isolated inflammatory signals or metabolic abnormalities alone, but rather arises from their mutual amplification. The significant associations observed for IVX and MMX individually, coupled with the superior predictive efficacy of MVX, suggest that inflammation and metabolic disturbances do not act in isolation but synergistically potentiate disease risk through positive feedback loops. RCS analysis further revealed significant linear dose-response relationships between MVX, IVX, MMX and the risk of SLE. Notably, the risk threshold for MVX was 36, which was lower than that for IVX (40) and MMX (45), suggesting that a combined inflammatory-metabolic state, even at a lower magnitude, confers a stronger disease risk than simple additive inflammation or metabolic disturbances alone. Furthermore, given that MVX was assessed only at baseline in this study, we evaluated potential bias by shortening the follow-up period. The results indicated only minimal regression dilution bias, and our overall conclusion regarding the significant association between MVX and SLE risk remained robust. MVX is a composite index derived from six metabolites; the utility of metabolites as long-term biomarkers for disease risk is well-established (11). Specifically, plasma BCAAs (valine, leucine, and isoleucine) have been shown to exhibit minimal fluctuation over periods spanning several decades (66). This stability explains why, in our study, various sensitivity analyses including a 3-year exclusion and landmark analyses at 5 and 10 years yielded a consistent association between MMX and SLE risk within a narrow range (HR: 1.24–1.28). Moreover, glycA is characterized by low intra-individual variability (67). Considering the stability of these circulating metabolites, combined with the analysis results of this study, MVX serves as a reliable reflection of long-term exposure to SLE.

Sex-stratified analyses revealed that the association between MVX and SLE was primarily evident in the female population. This finding aligns with the well-established female predominance in SLE incidence. Sex hormones play a critical role in regulating lipid metabolism, mitochondrial function, and inflammatory signaling (68), and a shift in the metabolic profile toward an adverse inflammatory–metabolic state after menopause has been demonstrated in prospective metabolomic studies (69). Thus, the significant predictive ability of MVX in women may partly reflect the complex interplay among sex hormones, metabolic status, and inflammatory networks. Furthermore, subgroup analyses demonstrated that the predictive value of MVX remained consistent across different BMI categories, smoking and drinking statuses, and physical activity levels, indicating its broad applicability as a risk marker.

This study has several limitations. First, the study population consisted predominantly of individuals of European ancestry; although the sample size was substantial, the generalizability of our findings to other ethnic groups, in whom SLE prevalence and severity may differ, requires further validation. Second, the metabolic biomarkers were measured at a single time point (baseline), precluding an assessment of longitudinal fluctuations in metabolic vulnerability and its impact on SLE risk. Finally, given the observational nature of the study, we can identify associations but cannot definitively establish causality or elucidate the precise molecular mechanisms underlying the observed relationships.

## 5 Conclusion

This study provides comprehensive evidence that MVX, a composite index integrating inflammatory susceptibility and metabolic malfunction, is significantly and independently associated with an increased risk of SLE. These findings underscore the importance of targeting metabolic-inflammatory pathways in the primary prevention and clinical management of SLE, paving the way for future research into precision medicine strategies based on metabolic profiling.

## Data Availability

The raw data supporting the conclusions of this article will be made available by the authors, without undue reservation.
