# Supplementary material for: Association of metabolic and inflammation vulnerabilities with systemic lupus erythematosus: a prospective UK Biobank study
Source: Front Immunol. 2026 May 8;17:1819233. doi: 10.3389/fimmu.2026.1819233 (PMC13195015; doi:10.3389/fimmu.2026.1819233)
Supplement: Supplementary file 1 [file Supplementaryfile1.docx]

**Supplementary Methods**

**Supplementary Methods 1: List of Variables and Corresponding UK Biobank Field IDs**

| **Category** | **Variable Name** | **UKB Field ID** |
| --- | --- | --- |
| Metabolic Biomarkers | Citrate | p23473 |
|  | Isoleucine | p23465 |
|  | GlycA | p234780 |
|  | Leucine | p23466 |
|  | Valine | p23467 |
|  | Small HDL particles | p23572 |
| Demographic Factors | Age | p21003 |
|  | Gender | p31 |
|  | Ethnicity | p21000 |
| Socioeconomic Indicators | Education attainment | p6138 |
|  | Household income | p378 |
|  | Townsend Deprivation Index (TDI) | p22189 |
| Lifestyle Factors | Smoking status | p20116 |
|  | Drinking status | p20117 |
|  | Physical activity | p884, p894, p904, p914 |
| Anthropometric Measures | BMI | p21001 |
| Biochemical Markers | Triglycerides | p30870 |
|  | Cholesterol | p30690 |
| Follow-up & Vital Status | Follow-up start date (Baseline visit) | p53 |
|  | Death | p40000 |
|  | Loss to follow-up | p191 |
| Clinical History | Cancer history | p20001 |
|  | Cardiovascular disease (CVD) history | p20002 |
|  | Chronic liver disease (CLD) history | p20002 |

**Supplementary Methods 2**

**Details of Baseline Exclusion**

At baseline, we excluded participants who were using hormones or immunosuppressants. Specifically, for the hormone category (p20003), individuals were excluded if they reported the use of Prednisolone (covering 5 coding events: 1140874930, 1140874976, 1140883026, 1141157402), Prednisone (1140868364), Methylprednisolone (covering 2 coding events: 1140874976, 1140883026), Dexamethasone (covering 5 coding events: 1140874816), or Hydrocortisone (1140875668), which resulted in the exclusion of 3,525 participants. Regarding immunosuppressants, we excluded those using Methotrexate (covering 2 coding events: 1140869848, 1140910036), Azathioprine (1140869930, 1140909864), Cyclophosphamide (1140869604), Mycophenolate (1140925978), Leflunomide (1141166294, 1141166302, 1141166304, 1141166306), Ciclosporin (1140909844, 1141181020), Tacrolimus (1140911642, 1141179842), Hydroxychloroquine (1140884308), or Sulfasalazine (1140909702).

**Supplementary Methods 3**

**Covariates assessment**

Cardiovascular disease (CVD) and chronic liver disease (CLD) status were determined using non-cancer diagnosis records (P20002) and the year of first non-cancer diagnosis (P20008). A history of CVD was defined as yes if the participant had any of the ICD-10 codes—specifically 1067, 1068, 1078, 1079, 1080, 1081, 1082, 1086, 1087, 1088, 1093, 1094, 1425, 1426, 1065, 1479, 1483, 1484, 1485, 1486, 1487, 1488, 1489, 1490, 1491, 1492, 1583, 1584, 1585, 1586, 1587, 1588, 1589, 1590, 1591, or 1592—recorded before baseline; otherwise, it was classified as no. Similarly, a positive history of CLD was assigned if any of the codes 1155, 1156, 1157, 1158, 1506, 1507, 1508, 1578, 1579, 1580, 1581, 1582, or 1604 were present in the pre-baseline records, and no otherwise. Furthermore, Cancer history (Cancer history) was determined based on cancer diagnosis reports (P20001) and the year of first cancer diagnosis (P20006). If a participant had any record of cancer diagnosis prior to the baseline, the variable was coded as yes; conversely, it was coded as no.

**Supplementary Methods 4**

**MVX calculation**

Values for each metabolic biomarkers were winsorized at the 1st and 99th percentile. The unified unit for the six biological markers is μmol/L. Multi-marker scores were normalized using min-max scaling to a range of 1 to 100 before analysis. The sex-specific equations are provided below, with reference to the CATHGEN biorepository[1].

**R script:**

df %>% mutate(

IVX = case_when(

sex == "F" ~ 9 - (GlycA * 0.000187) - (sHDL * 0.3585) + (GlycA * sHDL * 0.000348),

sex == "M" ~ 9 - (GlycA * 0.00437) - (sHDL * 0.52307) + (GlycA * sHDL * 0.000817)

),

MMX = case_when(

sex == "F" ~

((4 - (Leucine * 0.03142) + (Leucine^2 * 0.0000893)) * 0.353) +

((7 - (Valine * 0.03362) + (Valine^2 * 0.0000689)) * 0.684) +

(Isoleucine * 0.00332) +

((1 - (Citrate * 0.0072) + (Citrate^2 * 0.0000573)) * 0.7135),

sex == "M" ~

((4 - (Leucine * 0.01594) + (Leucine^2 * 0.0000291)) * 1.076) +

((7 - (Valine * 0.0239) + (Valine^2 * 0.00005)) * 0.414) +

(Isoleucine * 0.01265) +

((1 + (Citrate * 0.00906) - (Citrate^2 * 0.0000126)) * 0.5881)

),

MVX = case_when(

sex == "F" ~

(IVX * 2.27278) + (log(MMX) * 12.13511) - (IVX * log(MMX) * 1.09312),

sex == "M" ~

(IVX * 3.54601) + (log(MMX) * 14.41428) - (IVX * log(MMX) * 1.43438)

)

)

**For the female cohort:**

The IVX calculation formula is: 9 + (GlycA × -0.000187) + (sHDL × -0.3585) + ((GlycA × sHDL) × 0.000348).

The MMX calculation comprises two weighted components, as per the following formula:

MMX = ((4 + (Leu × -0.03142) + (Leu²) × 0.0000893) × 0.353) + ((7 + (Val × -0.03362) + (Val²) × 0.0000689) × 0.684) + (Ile × 0.00332) + ((1 + (Citr × -0.0072) + (Citr²) × 0.0000573)) × 0.7135.

The calculation logic for MVX is: (IVX × 2.27278) + (ln(MMX) × 12.13511) + (IVX × ln(MMX)) × -1.09312.

**For the male cohort:**

The IVX formula is: 9 + (GlycA × -0.00437) + (sHDL × -0.52307) + ((GlycA × sHDL) × 0.000817).

The MMX formula is adjusted to:

MMX = ((4 + (Leu × -0.01594) + (Leu²) × 0.0000291) × 1.076) + ((7 + (Val × -0.0239) + (Val²) × 0.00005) × 0.414) + (Ile × 0.01265) + ((1 + (Citr × 0.00906) + (Citr²) × -0.0000126)) × 0.5881.

The formula for calculating MVX is: (IVX × 3.54601) + (ln(MMX) × 14.41428) + (IVX × ln(MMX)) × -1.43438.

**References**

1. Otvos, J.D., et al., Multimarkers of metabolic malnutrition and inflammation and their association with mortality risk in cardiac catheterisation patients: a prospective, longitudinal, observational, cohort study. Lancet Healthy Longev, 2023. 4(2): p. e72-e82.
