# Supplementary material for: Association of metabolic and inflammation vulnerabilities with systemic lupus erythematosus: a prospective UK Biobank study
Source: Front Immunol. 2026 May 8;17:1819233. doi: 10.3389/fimmu.2026.1819233 (PMC13195015; doi:10.3389/fimmu.2026.1819233)
Supplement: Supplementary file 2 [file Supplementaryfile2.docx]

| Variable | Chi-square | df | p-value | Variable | Chi-square | df | p-value | Variable | Chi-square | df | p-value |
| --- | --- | --- | --- | --- | --- | --- | --- | --- | --- | --- | --- |
| Incident SLE | | | | | | | | | | | |
| MVX | 3.526 | 1 | 0.060 | IVX | 1.844 | 1 | 0.175 | MMX | 0.530 | 1 | 0.467 |
| Age | 10.052 | 1 | 0.002 | Age | 9.953 | 1 | 0.002 | Age | 9.948 | 1 | 0.002 |
| Ethnicity | 2.598 | 1 | 0.107 | Ethnicity | 2.550 | 1 | 0.110 | Ethnicity | 2.500 | 1 | 0.114 |
| Gender | 6.447 | 1 | 0.011 | Gender | 6.451 | 1 | 0.011 | Gender | 6.656 | 1 | 0.010 |
| Drinking status | 6.252 | 3 | 0.100 | Drinking status | 6.278 | 3 | 0.099 | Drinking status | 6.289 | 3 | 0.098 |
| Smoking status | 6.361 | 3 | 0.095 | Smoking status | 6.329 | 3 | 0.097 | Smoking status | 6.336 | 3 | 0.096 |
| TDI | 0.259 | 1 | 0.611 | TDI | 0.270 | 1 | 0.604 | TDI | 0.265 | 1 | 0.607 |
| BMI | 1.810 | 3 | 0.613 | BMI | 1.819 | 3 | 0.611 | BMI | 1.875 | 3 | 0.599 |
| Household income | 4.780 | 5 | 0.443 | Household income | 4.804 | 5 | 0.440 | Household income | 4.793 | 5 | 0.442 |
| Education | 0.497 | 2 | 0.780 | Education | 0.489 | 2 | 0.783 | Education | 0.492 | 2 | 0.782 |
| Physical activity | 4.801 | 2 | 0.091 | Physical activity | 4.851 | 2 | 0.088 | Physical activity | 4.819 | 2 | 0.090 |
| Cholesterol | 0.928 | 1 | 0.335 | Cholesterol | 0.956 | 1 | 0.328 | Cholesterol | 0.924 | 1 | 0.337 |
| Triglycerides | 1.967 | 1 | 0.161 | Triglycerides | 2.037 | 1 | 0.154 | Triglycerides | 1.842 | 1 | 0.175 |
| Cancer | 0.016 | 1 | 0.899 | Cancer | 0.021 | 1 | 0.886 | Cancer | 0.022 | 1 | 0.883 |
| CVD history | 6.484 | 1 | 0.011 | CVD history | 6.556 | 1 | 0.011 | CVD history | 6.513 | 1 | 0.011 |
| CLD history | 0.216 | 1 | 0.642 | CLD history | 0.197 | 1 | 0.658 | CLD history | 0.236 | 1 | 0.627 |
| T2DM history | 0.055 | 1 | 0.815 | T2DM history | 0.062 | 1 | 0.803 | T2DM history | 0.050 | 1 | 0.824 |
| Global Test | | | | | | | | | | | |
| Global | 57.049 | 29 | 0.002 | Global | 55.852 | 29 | 0.003 | Global | 55.914 | 29 | 0.003 |

**Supplementary Result 1: Testing the Proportional Hazards Assumption for Three Exposure Indicators and SLE Based on Schoenfeld Residuals**

Note: The proportional hazards assumption was evaluated using Schoenfeld residuals. A P-value > 0.05 indicates that the assumption is not violated.

Abbreviations: SLE: Systemic Lupus Erythematosus; TDI: Townsend Deprivation Index; CVD: Cardiovascular Disease; CLD: Chronic Liver Disease; T2DM: Type 2 Diabetes Mellitus.

| **Fine-Gray competing risk regression analysis for MVX and SLE** | | | | | | |
| --- | --- | --- | --- | --- | --- | --- |
| Competing Risks | Coef | SE | SHR | 95% CI | Z value | P value |
| All-cause mortality | 0.392 | 0.050 | 1.48 | 1.34-1.63 | 7.79 | <0.01 |
| CVD-specific mortality | 0.413 | 0.050 | 1.51 | 1.37-1.67 | 8.26 | <0.01 |

**Supplementary Result 2**

Note: Fine and Gray’s subdistribution hazard models were used. MVX indicates metabolic vulnerability index; SHR, subdistribution hazard ratio; CI, confidence interval; SE, standard error. Coef. represents the subdistribution hazard coefficient.

| **Supplementary Result 3: Baseline characteristics of participants with missing vs complete covariates undergoing multiple imputation** | | | |
| --- | --- | --- | --- |
| Characteristic | No Missing Data (N = 398,200) | Missing Data (N = 63,116) | P-value |
| **Age** |  |  | <0.001 |
| Median (Q1, Q3) | 57.00 (50.00, 63.00) | 57.00 (50.00, 63.00) |  |
| **Gender** |  |  | <0.001 |
| Female | 214,351 (53.83%) | 35,364 (56.03%) |  |
| Male | 183,849 (46.17%) | 27,752 (43.97%) |  |
| **Ethnicity** |  |  | <0.001 |
| Non White | 20,746 (5.21%) | 5,037 (7.98%) |  |
| White | 377,454 (94.79%) | 58,079 (92.02%) |  |
| **Drinking status** |  |  | <0.001 |
| Current | 367,300 (92.24%) | 59,531 (94.32%) |  |
| Never | 17,162 (4.31%) | 2,449 (3.88%) |  |
| Previous | 13,738 (3.45%) | 1,136 (1.80%) |  |
| **Smoking status** |  |  | 0.689 |
| Current | 42,209 (10.60%) | 6,533 (10.35%) |  |
| Never | 217,338 (54.58%) | 34,543 (54.73%) |  |
| Previous | 138,613 (34.81%) | 22,040 (34.92%) |  |
| **Tdi** |  |  | <0.001 |
| Median (Q1, Q3) | -2.17 (-3.66, 0.48) | -2.00 (-3.57, 0.86) |  |
| **BMI** |  |  | <0.001 |
| Normal | 129,375 (32.49%) | 20,879 (33.08%) |  |
| Obese | 97,240 (24.42%) | 16,246 (25.74%) |  |
| Overweight | 168,518 (42.32%) | 25,852 (40.96%) |  |
| Underweight | 3,066 (0.77%) | 139 (0.22%) |  |
| **Household income** |  |  | <0.001 |
| 18,000 to 30,999 | 102,218 (25.67%) | 16,309 (25.84%) |  |
| 31,000 to 51,999 | 100,705 (25.29%) | 16,290 (25.81%) |  |
| 52,000 to 100,000 | 77,410 (19.44%) | 12,162 (19.27%) |  |
| Greater than 100,000 | 20,467 (5.14%) | 3,238 (5.13%) |  |
| Less than 18,000 | 97,400 (24.46%) | 15,116 (23.95%) |  |
| **Education** |  |  | <0.001 |
| College | 129,017 (32.40%) | 23,757 (37.64%) |  |
| Other | 197,388 (49.57%) | 29,355 (46.51%) |  |
| Unknown | 71,795 (18.03%) | 10,004 (15.85%) |  |
| **CVD history** |  |  | <0.001 |
| No | 164,656 (41.35%) | 26,117 (41.38%) |  |
| Yes | 233,544 (58.65%) | 36,999 (58.62%) |  |
| **CLD history** |  |  | <0.001 |
| No | 394,736 (99.13%) | 62,535 (99.08%) |  |
| Yes | 3,464 (0.87%) | 581 (0.92%) |  |
| **T2DM history** |  |  | <0.001 |
| No | 335,006 (84.13%) | 54,868 (86.93%) |  |
| Yes | 63,194 (15.87%) | 8,248 (13.07%) |  |
| **Cancer history** |  |  | <0.001 |
| No | 290,049 (72.84%) | 45,273 (71.73%) |  |
| Yes | 108,151 (27.16%) | 17,843 (28.27%) |  |
| **Cholesterol (mmol/L)** |  |  | <0.001 |
| Median (Q1, Q3) | 4.68 (4.01, 5.58) | 4.65 (3.95, 5.50) |  |
| **Triglycerides (mmol/L)** |  |  | <0.001 |
| Median (Q1, Q3) | 1.88 (1.30, 2.65) | 1.92 (1.35, 2.70) |  |

Note: Continuous variables are presented as median (interquartile range [Q1, Q3]), and categorical variables are presented as number (percentage). The differences between groups were compared using the Wilcoxon rank-sum test for continuous variables and the Chi-square test for categorical variables.

**Supplementary Result 4**

**The results of the variance inflation factors GVIF tests for the model variables**

| **MVX and SLE** | | | |
| --- | --- | --- | --- |
| **Variable** | **GVIF** | **DF** | **GVIF^(1/(2*Df))** |
| MVX (per SD) | 1.71 | 1 | 1.310 |
| Drinking status | 47.20 | 3 | 1.901 |
| Smoking status | 46.70 | 3 | 1.898 |
| Physical activity | 11.99 | 2 | 1.861 |
| ethnicity | 6.93 | 2 | 1.622 |
| Household income | 7.15 | 5 | 1.217 |
| Age | 1.33 | 1 | 1.153 |
| Triglycerides | 1.26 | 1 | 1.125 |
| TDI | 1.18 | 1 | 1.088 |
| Cholesterol | 1.20 | 1 | 1.094 |
| education | 1.33 | 2 | 1.074 |
| gender | 1.16 | 1 | 1.079 |
| CVD history | 1.16 | 1 | 1.077 |
| T2DM_history | 1.06 | 1 | 1.028 |
| BMI | 1.22 | 3 | 1.033 |
| Cancer | 1.02 | 1 | 1.010 |
| CLD history | 1.00 | 1 | 1.001 |

Abbreviations: GVIF: Generalized Variance Inflation Factor. DF: degrees of freedom. GVIF^(1/(2*Df)) value < 5 suggests no significant multicollinearity

**Supplementary Result 5**

| **Changes in the association between MVX and SLE risk after sequentially excluding individual metabolites (leave-one-out approach)** | | | | | | | | | | | | |
| --- | --- | --- | --- | --- | --- | --- | --- | --- | --- | --- | --- | --- |
|  | Replacing with Mean | Case N | Control N | Model 1 | | | Model 2 | | | Model 3 | | |
|  |  |  |  | HR | 95%CI | Pvalue | HR | 95%CI | Pvalue | HR | 95%CI | Pvalue |
| MVX (per SD) | NO | 408 | 397,792 | 1.41 | 1.27-1.56 | ＜0.01 | 1.46 | 1.30-1.64 | ＜0.01 | 1.44 | 1.28-1.62 | ＜0.01 |
| MVX (per SD) | GlycA | 408 | 397,792 | 1.29 | 1.18-1.42 | ＜0.01 | 1.29 | 1.17-1.42 | ＜0.01 | 1.29 | 1.17-1.42 | ＜0.01 |
| MVX (per SD) | sHDL | 408 | 397,792 | 1.37 | 1.24-1.51 | ＜0.01 | 1.42 | 1.28-1.59 | ＜0.01 | 1.40 | 1.25-1.56 | ＜0.01 |
| MVX (per SD) | Leucine | 408 | 397,792 | 1.30 | 1.18-1.44 | ＜0.01 | 1.35 | 1.20-1.52 | ＜0.01 | 1.33 | 1.18-1.50 | ＜0.01 |
| MVX (per SD) | Valine | 408 | 397,792 | 1.32 | 1.19-1.46 | ＜0.01 | 1.39 | 1.24-1.57 | ＜0.01 | 1.37 | 1.21-1.54 | ＜0.01 |
| MVX (per SD) | Isoleucine | 408 | 397,792 | 1.42 | 1.28-1.56 | ＜0.01 | 1.44 | 1.30-1.61 | ＜0.01 | 1.42 | 1.28-1.58 | ＜0.01 |
| MVX (per SD) | Citrate | 408 | 397,792 | 1.41 | 1.27-1.57 | ＜0.01 | 1.46 | 1.30-1.64 | ＜0.01 | 1.43 | 1.26-1.61 | ＜0.01 |

Model 1 was adjusted for age, gender, and ethnicity. Model 2 was further adjusted for drinking status, smoking status, TDI, BMI, household income, education, and physical activity. Model 3 was additionally adjusted for cancer history, T2DM history, history of cardiovascular disease, history of chronic liver disease, and lipid levels. All covariates were assessed at baseline. P-values were two-sided, with statistical significance defined as P < 0.05.

Abbreviations: HR: Hazard ratio; CI: Confidence interval; TDI: Townsend Deprivation Index; Ref: Reference group; MVX: Metabolic Vulnerability Index; IVX: Inflammation Vulnerability Index; MMX: Metabolic Malnutrition Index; SD: Standard Deviation; CRP: C-Reactive Protein.
